# Presentations and management of hospitalized patients with upper extremity fractures at a level 1 trauma center: a 5-year observational study

**DOI:** 10.1007/s00590-024-04017-2

**Published:** 2024-06-17

**Authors:** Syed Imran Ghouri, Mohammad Asim, Ayman El-Menyar, Ibrahim Afifi, Yassir Abdulrahman, Hisham Jogol, Hassan Al-Thani, Sandro Rizoli

**Affiliations:** 1 Department of Surgery, Orthopedic Surgery, Hamad Medical Corporation, P.O. Box 3050, Doha, Qatar; 2 Department of Surgery, Clinical Research, Trauma and Vascular Surgery, Hamad Medical Corporation, Doha, Qatar; 3 Department of Clinical Medicine, Weill Cornell Medical College, P.O. Box 24144, Doha, Qatar; 4 Department of Surgery, Trauma Surgery, Hamad General Hospital, Doha, Qatar; 5 Department of Surgery, Trauma and Vascular Surgery, Hamad Medical Corporation, Doha, Qatar

**Keywords:** Upper extremity injury, Mechanism of injury, Trauma, Orthopedics, Qatar

## Abstract

**Background:**

Upper extremity injuries (UEIs) are common in the emergency departments, yet they are under-reported in developing countries. This study examined the frequency, injury characteristics, and treatment approaches of upper extremity fractures (UEFs) among hospitalized trauma patients in a nationally representative population.

**Methods:**

We conducted a retrospective, observational study including all the hospitalized patients with UEFs in the only level 1 trauma center in Qatar between July 2015 and August 2020. Comparative analyses were performed according to injury mechanisms, severity, and management approach.

**Results:**

A total of 2,023 patients sustained UEIs with an average age of 34.4 ± 12.9 years, and 92% were males. Motor vehicle crashes (MVCs; 42.3%) were the primary cause of shoulder girdle injuries in 48.3% of cases. Fractures of the radius, ulna, and hands occurred in 30.8, 16.5 and 14.5%, respectively. Young adults were more involved in MVCs and motorcycle crashes (MCCs), while pedestrians who were typically older had a higher rate of humerus fractures. Patients with MCCs had a higher rate of clavicle and ulna fractures. Pedestrians were at risk of serious injuries, with a higher mean injury severity score and lower Glasgow Coma Scale.

**Conclusion:**

Most UEFs patients were young males and mainly affected by MVCs. Shoulder girdle, particularly clavicle and scapula/glenoid fractures, emerged as common injury sites. The study highlighted the potential risk of pedestrian injuries, as reflected in higher injury severity, concomitant injuries, and higher mortality. Future studies are needed to optimize preventive measures by incorporating insights into specific injury mechanisms and patterns of UEIs.

## Introduction

Injuries to the extremities continue to be a significant healthcare issue in most countries, which affects the quality of life (QoL). Therefore, understanding the patterns of upper limb (UL) injuries is crucial for effective treatment. Upper extremity injuries (UEIs) are common injuries in the emergency department (ED) across the globe [1, 2]. These may vary in severity from simple fingertips to complicated injuries involving several UL structures [3]. Traumatic fractures of the upper extremities commonly manifest as standalone injuries; however, in polytrauma patients, up to 30% of cases had UEIs in the presence of other concomitant injuries [4]. Notably, severe injuries to the upper extremities, such as amputation, brachial plexus damage, and severely damaged limbs, necessitate a comprehensive, multidisciplinary management approach for successful reconstruction [5, 6]. Of note, inadequate handling of injuries to the upper extremities may lead to significant impairment that affects the functional outcomes [7]. Complicated fracture patterns, polytrauma, delayed management, thin layers of soft tissue, and an inadequate vascular supply are associated with poor outcomes [8], including the QoL.

The epidemiological characteristics of UEFs differ across countries and even within regions, which is often influenced by socioeconomic factors, occupation, and recreational activities [9]. Karl et al. [10] reported an annual incidence of 67.6 UEFs per 10,000 individuals in 2009 in the United States. An Italian report highlighted 201,940 hospitalizations with an incidence rate of 340 per 100,000 persons/year [11], while in the Netherlands, the incidence increased by 13% from 1986 to 2008 [12]. However, in recent years, there has been a decline in the number of cases in developed countries.

Contrary to the global trend of decline, the MENA region (Middle East and North Africa) has shown a 2.3% increase in the age-adjusted incidence rate of UEFs over the past three decades, while the disability rate has surged by 15.6% [13]. Despite the high prevalence of UEIs, most existing literature focuses on lower extremity trauma, resulting in a significant gap in understanding the epidemiology and outcomes of UEFs, particularly among hospitalized trauma patients in developing countries. Therefore, we aim to outline the incidence, injury characteristics, and treatment approaches of UEFs, considering injury mechanisms and severity, in a nationally representative population in Qatar.

## Methods

We conducted a retrospective, observational study that included all patients with UEIs admitted to the only level 1 trauma center at Hamad Trauma Center (HTC) in Qatar from July 2015 to August 2020. The HTC, the sole tertiary facility in the country, annually admits and treats approximately 2000 patients with moderate-to-severe injuries. Upon arrival at HTC, patients are evaluated and managed according to the advanced trauma life support (ATLS) guidelines. Data were retrieved from the Qatar National Trauma Registry (QNTR) and the electronic medical records. The QNTR contributes to the American College of Surgeons (ACS) Committee on Trauma's National Trauma Databank (NTDB). The trauma registry undergoes international and local validations with quarterly reports to the NTDB and ACS-TQIP [14, 15]. Patients with incomplete data, treated and discharged from the ED, those who were dead on arrival or before management, and patients who were transferred to other facilities were excluded.

Collected data included patient demographics, mechanism of injury (MOI), ED disposition, vital signs, type of injury (open/closed), anatomical location of UEIs, associated injuries, injury severity score (ISS), Glasgow coma score (GCS), abbreviated injury scores, management (surgical or conservative), blood transfusion, hospital length of stay, in-hospital complications, and mortality.

### Statistical analysis

Data were expressed as numbers, percentages, and mean ± standard deviation or medians and ranges, whenever appropriate. Comparative analyses were performed for subgroups according to mechanisms of injury; severity of injury [mild (ISS ≤ 8), moderate (ISS 9–15), severe (ISS 16–25), and critical (ISS > 25)]; and management (conservative vs. surgical). The chi-square test was performed to analyze differences in categorical variables between groups, and the Fisher exact test was used when cell values n < 5. Continuous variables were analyzed using Student's t-test for two groups or ANOVA test for > two groups. Mann–Whitney U and Kruskal–Wallis tests were used for non-parametric data whenever applicable. Two-tailed p < 0.05 were considered significant. Data was analyzed using the Statistical Package for the Social Sciences version 22.0 (SPSS Inc. Chicago, Illinois, USA).

## Results

**Fig. 1:**
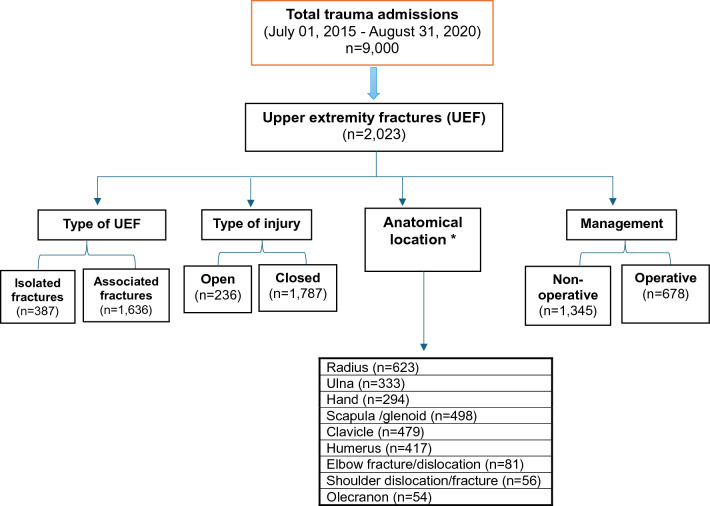
Cohort distribution (*there is an overlap for different types of fractures)

**Fig. 2:**
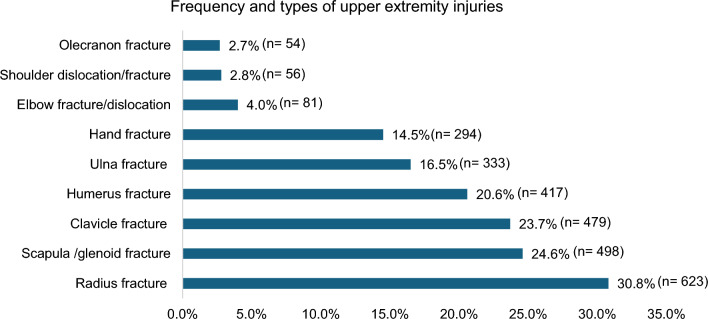
Frequency and types of upper extremity injuries among hospitalized trauma patients over 5 years

During the study period, 2,023 patients sustained UEFs requiring hospitalization with an average age of 34.4 ± 12.9 years. The majority were male (92%). The most frequent MOI was motor vehicle crashes (42.3%), followed by falls from height (27.1%), pedestrian incidents (12.5%), and motorcycle crashes (8.5%). From the emergency department, less than half of injured patients were transferred to the ward (46.8%), while 30% required intensive care unit admission and 17.8% necessitated immediate surgical intervention. Ethanol was positive in 7.2% of cases. Most injuries were closed (88.3%), with only 11.7% being open fractures (Fig. 1). Approximately half of the injuries involved the shoulder girdle (48.3%), including clavicle (23.7%), scapula, and glenoid (24.6%) fracture or dislocation (Fig. 2). Humeral shaft fracture accounted for 20.6%, and fracture or dislocation of the elbow was observed in 4%. Fractures of the radius (30.8%) and ulna (16.5%) were frequent, followed by hand fractures (14.5%).

Chest injuries were the most associated injury (52.9%), followed by head injuries (30.4%), lower extremities (29.3%), abdomen (20.9%) and pelvis injuries (20.3%). The median ISS was 14 (9–22), with most cases being moderate (36.6%) to severe (26.7%). The mean upper extremity AIS was 2.0 ± 0.9, and the lower extremity AIS was 2.5 ± 0.5. In 33.5% of cases, surgical intervention was required, and 27.8% needed blood transfusion. Few patients developed pneumonia (3.9%), sepsis (1.4%), and ARDS (1.3%). The median hospital and ICU length of stay were 4 (1–115) and 7 (1–505) days, respectively. The overall in-hospital mortality rate was 7.5%, affecting 52 polytrauma patients.

Table 1 compares demographics, clinical characteristics, and outcomes based on the MOI. Younger adults were more frequently involved in motor vehicle and motorcycle crashes, while injuries caused by pedestrian accidents more frequently happened in relatively older individuals (*p* = 0.001). Notably, open fractures were more frequent among the fall victims (*p* = 0.001), with specific injuries such as radius (*p* = 0.001), olecranon (*p* = 0.001), and hand (*p* = 0.001) fractures. Humerus fractures were significantly higher in the pedestrian group (*p* = 0.002). Motorcycle crashes were associated with an increased likelihood of clavicle and ulna fractures (*p* = 0.001). In contrast, scapula or glenoid fractures were more prevalent in patients injured by falls of heavy objects (*p* = 0.001). Pedestrians were at risk of severe injuries with higher mean ISS, lower GCS (*p* = 0.001), and a higher need for blood transfusions (*p* = 0.001).

**Table 1:** demographics, clinical characteristics, and outcomes of patients who sustained upper extremity injuries based on the mechanism of injury

	Motor vehicle crash (n = 856)	Fall (n = 548)	Pedestrian (n = 253)	Motorcycle crash (n = 171)	Fall of heavy object (n = 84)	Others (n = 111)	*p* value
Age	32 ± 13	37 ± 13	39 ± 14	31 ± 10	34 ± 8	33 ± 10	0.001
Males	755 (88.2%)	524 (95.6%)	236 (93.3%)	169 (98.8%)	84 (100%)	97 (87.4%)	0.001
Ethanol positive	84 (9.8%)	15 (2.7%)	19 (7.5%)	16 (9.4%)	0 (0.0%)	12 (10.8%)	0.001
Ethanol levels	31.8 ± 14.3	41.4 ± 14.3	39.4 ± 20.8	43.1 ± 14.5	-	20.7 ± 14.7	0.001
*Type of injury*							
Open	88 (10.3%)	74 (13.5%)	20 (7.9%)	20 (11.7%)	10 (11.9%)	24 (21.6%)	0.004 for all
Close	768 (89.7%)	474 (86.5%)	233 (92.1%)	151 (88.3%)	74 (88.1%)	87 (78.4%)	
Upper extremity injuries							
Clavicle fracture	247 (28.9%)	72 (13.1%)	72 (28.5%)	55 (32.2%)	23 (27.4%)	10 (9.0%)	0.001
Scapula /glenoid fracture	220 (25.7%)	111 (20.3%)	78 (30.8%)	33 (19.3%)	37 (44.0%)	19 (17.1%)	0.001
Shoulder dislocation/fracture	24 (2.8%)	14 (2.6%)	9 (3.6%)	4 (2.3%)	2 (2.4%)	3 (2.7%)	0.97
Humerus fracture	194 (22.7%)	94 (17.2%)	67 (26.5%)	28 (16.4%)	9 (10.7%)	25 (22.5%)	0.002
Elbow fracture/dislocation	26 (3.0%)	33 (6.0%)	8 (3.2%)	5 (2.9%)	4 (4.8%)	5 (4.5%)	0.10
Olecranon fracture	20 (2.3%)	26 (4.7%)	4 (1.6%)	1 (0.6%)	1 (1.2%)	2 (1.8%)	0.01
Radius fracture	203 (23.7%)	250 (45.6%)	50 (19.8%)	67 (39.2%)	18 (21.4%)	35 (31.5%)	0.001
Ulna fracture	139 (16.2%)	84 (15.3%)	28 (11.1%)	37 (21.6%)	9 (10.7%)	36 (32.4%)	0.001
Hand fracture	122 (14.3%)	80 (14.6%)	21 (8.3%)	25 (14.6%)	9 (10.7%)	37 (33.3%)	0.001
*Associated injuries*							
Head	253 (29.6%)	177 (32.3%)	102 (40.3%)	54 (31.6%)	10 (11.9%)	18 (16.2%)	0.001
Chest	511 (59.7%)	241 (44.0%)	151 (59.7%)	86 (50.3%)	54 (64.3%)	28 (25.2%)	0.001
Abdomen	200 (23.4%)	94 (17.2%)	68 (26.9%)	31 (18.1%)	18 (21.4%)	12 (10.8%)	0.001
Pelvis	170 (19.9%)	120 (21.9%)	71 (28.1%)	25 (14.6%)	17 (20.2%)	7 (6.3%)	0.001
Lower extremity	254 (29.7%)	124 (22.6%)	104 (41.1%)	58 (33.9%)	28 (33.3%)	24 (21.6%)	0.001
Injury severity score	14 (9–22)	14 (9–19)	17 (10–29)	14 (9–22)	14 (9–17)	11 (10–17)	0.001
GCS ED	15 (14–15)	15 (15–15)	15 (3–15)	15 (15–15)	15 (15–15)	15 (15–15)	0.001
Head AIS	3.4 ± 1.0	3.9 ± 0.9	3.7 ± 1.0	3.4 ± 0.9	3.1 ± 0.7	3.4 ± 0.6	0.03
Chest AIS	2.7 ± 0.7	2.6 ± 0.7	2.9 ± 0.7	2.7 ± 0.7	2.6 ± 0.7	2.6 ± 0.9	0.06
Abdomen AIS	2.6 ± 0.9	2.5 ± 0.8	2.6 ± 0.9	2.4 ± 0.8	2.6 ± 0.9	2.7 ± 1.2	0.84
Pelvis AIS	2.2 ± 0.6	2.1 ± 0.5	2.4 ± 0.8	2.6 ± 0.9	2.5 ± 1.0	2.7 ± 0.9	0.005
Lower extremity AIS	2.6 ± 0.5	2.6 ± 0.5	2.4 ± 0.5	2.6 ± 0.5	2.7 ± 0.5	2.6 ± 0.6	0.02
Upper extremity AIS	2.0 ± 0.3	2.0 ± 0.3	1.9 ± 0.2	2.0 ± 0.3	2.0 ± 0.4	2.1 ± 0.6	0.01
Surgical intervention	266 (31.1%)	223 (40.7%)	56 (22.1%)	70 (40.9%)	18 (21.4%)	45 (40.5%)	0.001
Blood transfusion	262 (30.6%)	109 (19.9%)	93 (36.8%)	42 (24.6%)	22 (26.2%)	34 (30.6%)	0.001
Number of blood units	4 (1–41)	4 (1–42)	5 (1–52)	4 (1–23)	4.5 (1–53)	4.5 (2–32)	0.82
*In-hospital complications*							
Pneumonia	39 (4.6%)	17 (3.1%)	13 (5.1%)	6 (3.5%)	2 (2.4%)	1 (0.9%)	0.28
Sepsis	14 (1.6%)	8 (1.5%)	4 (1.6%)	1 (0.6%)	1 (1.2%)	1 (0.9%)	0.92
ARDS	13 (1.5%)	4 (0.7%)	6 (2.4%)	1 (0.6%)	2 (2.4%)	1 (0.9%)	0.37
Ventilatory days	6 (1–115)	5 (1–43)	5 (1–29)	6 (1–51)	4.5 (1–30)	2 (1–12)	0.12
ICU length of stay	5 (1–115)	4 (1–76)	4 (1–45)	4 (1–58)	5 (1–74)	3.5 (1–32)	0.02
Hospital length of stay	8 (1–169)	6 (1–181)	6 (1–505)	6 (1–122)	5 (1–336)	6 (1–99)	0.002
Mortality	53 (6.2%)	33 (6.0%)	49 (19.4%)	10 (5.8%)	2 (2.4%)	5 (4.5%)	0.001

*AIS* abbreviated injury score, *ED* emergency department, *GCS* Glasgow coma scale, *ARDS* acute respiratory distress syndrome

Table 2 demonstrates the characteristics and outcomes of UEFs based on injury severity, categorized by the ISS. Patients with moderate-to-severe injuries were, on average, two years older than those with mild injuries (*p* = 0.001). Moderate injuries were commonly associated with MVCs, while pedestrians were more likely to sustain critical injuries. A higher proportion of motorcyclists sustained mild injuries (*p* = 0.001). The groups with severe and critical injuries had a significantly higher proportion of clavicle (*p* = 0.001) and scapula or glenoid (*p* = 0.001) fractures.

**Table 2:** demographics, clinical characteristics, and outcome of patients who sustained upper extremity injuries based on injury severity score

	Mild (ISS ≤ 8) (n = 394)	Moderate (ISS 9–15) (n = 734)	Severe (ISS 16–25) (n = 535)	Critical (ISS > 25) (n = 343)	*p* value
Age	32.7 ± 12.2	35.4 ± 13.6	34.9 ± 12.6	33.4 ± 13.1	0.001
Males	362 (91.9%)	670 (91.3%)	498 (93.1%)	319 (93.0%)	0.61
*Mechanism of injury*					
Motor vehicle crash	156 (39.6%)	312 (45.5%)	228 (42.6%)	155 (18.2%)	0.001 for all
Fall from height	114 (28.9%)	209 (28.5%)	152 (28.4%)	72 (21.0%)	
Pedestrian	37 (9.4%)	74 (10.1%)	66 (12.3%)	69 (20.1%)	
Motorcycle crash	37 (9.4%)	62 (8.4%)	42 (7.9%)	28 (8.2%)	
Fall of a heavy object	15 (3.8%)	33 (4.5%)	27 (5.0%)	8 (2.3%)	
Others	35 (8.9%)	44 (6.0%)	20 (3.7%)	11 (3.2%)	
*Type of injury*					
Open	56 (14.2%)	82 (11.2%)	51 (9.5%)	45 (13.1%)	0.12
Close	338 (85.5%)	652 (88.8%)	484 (90.5%)	298 (86.9%)	
*Upper extremity injuries*					
Clavicle fracture	50 (12.7%)	169 (23.0%)	162 (30.3%)	96 (28.0%)	0.001
Scapula /glenoid fracture	46 (11.7%)	157 (21.4%)	177 (33.1%)	118 (34.4%)	0.001
Shoulder dislocation/fracture	13 (3.3%)	19 (2.6%)	11 (2.1%)	11 (3.2%)	0.62
Humerus fracture	91 (23.1%)	137 (18.7%)	98 (18.3%)	83 (24.2%)	0.05
Elbow fracture/dislocation	24 (6.1%)	31 (4.2%)	14 (2.6%)	10 (2.9%)	0.03
Olecranon fracture	5 (1.3%)	23 (3.1%)	15 (2.8%)	11 (3.2%)	0.26
Ulna fracture	82 (20.8%)	122 (16.6%)	76 (14.2%)	53 (15.5%)	0.05
Radius fracture	154 (39.1%)	238 (32.4%)	137 (25.6%)	93 (27.1%)	0.001
Hand fracture	90 (22.8%)	104 (14.2%)	52 (9.7%)	47 (13.7%)	0.001
*Associated injuries*					
Head	9 (2.3%)	88 (12.0%)	262 (49.0%)	251 (73.2%)	0.001
Chest	33 (8.4%)	350 (47.7%)	392 (73.3%)	290 (84.5%)	0.001
Abdomen	11 (2.8%)	62 (8.4%)	172 (32.1%)	178 (51.9%)	0.001
Pelvis	53 (13.5%)	109 (14.9%)	114 (21.3%)	129 (37.6%)	0.001
Lower extremity	75 (19.0%)	216 (29.4%)	154 (28.8%)	139 (40.5%)	0.001
GCS ED (median, IQR)	15 (15–15)	15 (15–15)	15 (14–15)	Z`	0.001
Head AIS	1.8 ± 0.4	2.6 ± 0.6	3.0 ± 0.6	4.2 ± 0.9	0.001
Chest AIS	1.4 ± 0.5	2.5 ± 0.7	2.7 ± 0.6	3.2 ± 0.7	0.001
Abdomen AIS	1.7 ± 0.5	2.0 ± 0.4	2.3 ± 0.6	2.9 ± 1.0	0.001
Pelvis AIS	2.0 ± 0.0	2.0 ± 0.2	2.2 ± 0.5	2.6 ± 0.9	0.001
Lower extremity AIS	1.9 ± 0.2	2.6 ± 0.5	2.5 ± 0.5	2.6 ± 0.5	0.001
Upper extremity AIS	1.9 ± 0.2	2.0 ± 0.3	2.1 ± 0.4	2.1 ± 0.4	0.001
Surgical intervention	173 (43.9%)	248 (33.8%)	167 (31.2%)	90 (26.2%)	0.001
Blood transfusion	18 (4.6%)	125 (17.0%)	174 (32.5%)	245 (71.4%)	0.001
Number of blood units	2 (1–9)	3 (1–32)	4 (1–29)	7 (1–53)	0.001
*In-hospital complications*					
Pneumonia	1 (0.3%)	1 (0.1%)	22 (4.1%)	54 (15.7%)	0.001
Sepsis	1 (0.3%)	1 (0.1%)	7 (1.3%)	20 (5.8%)	0.001
ARDS	0 (0.0%)	0 (0.0%)	7 (1.3%)	20 (5.8%)	0.001
Ventilatory days	1 (1–28)	1 (1–12)	5 (1–97)	8 (1–115)	0.001
ICU LOS	1.5 (1–62)	2 (1–76)	4 (1–112)	11 (1–115)	0.001
Hospital LOS	3 (1–67)	5 (1–505)	10 (1–181)	18 (1–336)	0.001
Mortality	9 (2.3%)	15 (2.0%)	16 (3.0%)	95 (27.7%)	0.001

In contrast, the mild injury group was more likely to have elbow fracture or dislocation (*p* = 0.03), radius (*p* = 0.001), and hand fractures (*p* = 0.001).

Table 3 shows the clinical characteristics based on the management approach (surgical vs non-surgical). The two groups were comparable in terms of age and gender. Patients who underwent surgery had a significantly higher rate of open fractures (25.8% vs. 4.5%; *p* = 0.001). Clavicle fractures (*p* = 0.001) and scapula or glenoid fractures (*p* = 0.001) were more commonly treated conservatively. Head and chest trauma were more common in the conservative group (*p* = 0.001). The average upper and lower extremity AIS were higher in the surgical group (*p* = 0.001). The conservative group had longer ICU stays and higher in-hospital mortality rates (*p* = 0.001).

**Table 3:** clinical characteristics based on management approach

	Conservative (n = 1345)	Surgical intervention (n = 678)	*P* value
Age	34.9 ± 13.7	33.2 ± 11.4	0.003
Males	1246 (92.6%)	619 (91.3%)	0.28
Type of injury			
Close	1284 (95.5%)	503 (74.2%)	0.001 for all
Open	61 (4.5%)	175 (25.8%)	
*Upper extremity injuries*			
Clavicle fracture	429 (31.9%)	50 (7.4%)	0.001
Scapula /glenoid fracture	432 (32.1%)	66 (9.7%)	0.001
Shoulder dislocation/fracture	39 (2.9%)	17 (2.5%)	0.61
Humerus fracture	188 (14.0%)	229 (33.8%)	0.001
Elbow fracture/dislocation	44 (3.3%)	37 (5.5%)	0.01
Olecranon fracture	19 (1.4%)	35 (5.2%)	0.001
Ulna fracture	106 (7.9%)	227 (33.5%)	0.001
Radius fracture	237 (17.6%)	386 (56.9%)	0.001
Hand fracture	194 (14.4%)	100 (14.7%)	0.84
*Associated injuries*			
Head	463 (34.4%)	151 (22.3%)	0.001
Chest	798 (59.3%)	273 (40.3%)	0.001
Abdomen	283 (21.0%)	140 (20.6%)	0.83
Pelvis	259 (19.3%)	151 (22.3%)	0.11
Lower extremity	341 (25.4%)	251 (37.0%)	0.001
Injury severity score	17.3 ± 10.6	14.8 ± 9.7	0.001
GCS ED (median, IQR)	15 (12–15)	15 (15–15)	0.001
Head AIS	3.5 ± 0.9	3.3 ± 0.9	0.11
Chest AIS	2.8 ± 0.7	2.6 ± 0.8	0.01
Abdomen AIS	2.6 ± 0.9	2.5 ± 0.9	0.42
Pelvis AIS	2.3 ± 0.7	2.1 ± 0.5	0.02
Lower extremity AIS	2.5 ± 0.6	2.6 ± 0.5	0.003
Upper extremity AIS	1.9 ± 0.3	2.1 ± 0.4	0.001
Blood transfusion	339 (25.2%)	223 (32.9%)	0.001
Number of blood units	4 (1–53)	4 (1–42)	0.03
*In-hospital complications*			
Pneumonia	58 (4.3%)	20 (2.9%)	0.13
Sepsis	20 (1.5%)	9 (1.3%)	0.77
ARDS	22 (1.6%)	5 (0.7%)	0.09
Ventilatory days	5 (1–115)	5 (1–97)	0.99
ICU LOS	5 (1–115)	4 (1–112)	0.02
Hospital LOS	6 (1–505)	10 (1–134)	0.001
Mortality	150 (11.2%)	2 (0.3%)	0.001

## Discussion

During a 5-year study period, almost a quarter of trauma hospitalizations involved UEFs, predominantly affecting young males, who are mainly injured by motor vehicle and motorcycle crashes. Most patients sustained closed injuries, with shoulder girdle injuries being the most common (48.3%). Pedestrians were found to have a higher risk of severe injuries, characterized by higher mean ISS, lower GCS, and a frequent need for blood transfusions. Furthermore, severe and critically injured patients were more likely to have clavicle and scapula or glenoid fractures, leading to prolonged hospital course, increased in-hospital complications, and mortality rates. Approximately one-third of patients required surgical intervention, predominantly for open injuries involving fractures of the humerus, elbow, olecranon, ulna, radius, or hand.

A study from Italy [11] reported a prevalence of UEIs to be 20% among all emergency visits. However, another study from the Netherlands reported a higher occurrence of UEIs (42%) among trauma-related ED visits [12]. The predominance of UEIs in young men compared to women of the same age aligns with previous studies attributing this to recreational activities and high-velocity forces like road traffic accidents. This might be explained by the fact that young individuals are more impulsive, and that motorization is higher in rapidly developing high-income countries, including Qatar. Similar gender disparities were noted in other studies; for instance, Ameri et al. [9] demonstrated a predominance of males (77.5%), possibly linked to a higher population of young, active males in the country engaged in occupational/constructional activities and riskier behaviors. Another prior study conducted in the United States reviewed 90 million injuries presented to the ED, revealing that 35% involved the upper extremity [16]. Another study of 24,885 polytrauma patients reported extremity injuries in 39.7% of cases, with the most prevalent fractures being those of the femur (16.5%), tibia (12.6%), and clavicle (10.4%) [4]. In our cohort, most patients experienced closed injuries (88.3%), with shoulder girdle injuries being the most prevalent (48.3%). This was followed by fractures in the radius (30.8%) and humeral shaft (20.6%). A previous study analyzing 54,076 polytrauma cases (ISS ≥ 16) identified shoulder injuries in 27.9%, with 68.5% accounting for blunt trauma [17]. The higher occurrence of fractures in the shoulder and upper arms can be attributed to MVCs, which are the most frequent cause. During MVC, patients often experience high-impact forces, particularly on an outstretched hand, increasing the likelihood of shoulder and upper arm fractures or dislocations. Ribak et al. [18] reported a more significant occurrence of closed fractures, with a higher frequency of severe injuries associated with hand trauma. Another study identified fingers (38.4%) and wrists (15.2%) as the most frequently injured anatomical regions in the Mexican population [19]. Similarly, a study from the United States reported finger injuries (38.4%) to be most frequent, followed by shoulder (16.8%) [20]. We observed hand fractures in approximately 15% of cases, a finding similar to van Olsen and colleagues’ study (19%) [21].

In the current study, the highest prevalence of associated injuries was in the chest (52.9%), followed by the head (30.4%). A prior study reported head/face/neck injuries (52%) as common, followed by lower extremities (49%) and chest (46%) injuries [22]. In line with our findings, Briese et al. [17] indicated a correlation between shoulder injuries and trauma to the chest, spine, and head. Our study revealed a distinct pattern where younger males were disproportionately involved in MVC and motorcycle crashes, contrasting with pedestrian accidents, which are more prevalent among older adults. In adult car occupants, the most common open fractures were reported to be ulna (25%), radius (24%), and humerus (21%) [22]. However, in our study, clavicle (28.9%), scapula or glenoid (25.7%), and radius fractures (23.7%) were the prevalent types among MVC victims.

Another study focusing on the elderly suggested that pedestrians are more susceptible to bony injuries, including fractures of the lower and upper extremities, possibly due to age-related factors like osteoporosis, muscle dystrophy, and reduced subcutaneous tissue [23]. This suggests that age plays a role in influencing the nature and severity of pedestrian injuries.

In this study, the rate of associated injuries was disproportionately higher in severely injured patients, with distinct patterns based on injury mechanisms. For instance, humeral shaft fractures in MVC indicate increased risks of additional upper and lower extremity injuries and serve as predictors for concomitant abdominal injuries, notably liver injuries [24]. The severity of injuries caused by high-energy impact or force involved in the injury mechanism contributes to a diverse range of associated injuries in the prominent anatomical regions.

In our cohort, patients with humerus fractures, elbow fractures/dislocations, and fractures of the olecranon, ulna, radius, and hand often underwent surgical intervention. In contrast, clavicle and scapula or glenoid fractures were mainly managed conservatively. Consistent with our findings, Briese et al. [17] noted a significant increase in the surgical treatment rate with the injury's severity. The authors reported that surgical treatment was frequently considered for proximal humerus fractures and injured vessels, while scapula and clavicle fractures were more often managed conservatively.

The prolonged hospitalization can be attributed in part to the higher proportion of associated injuries and complications in severely injured patients with UEIs in our cohort. Similar results were observed by Zeelenberg et al. [25], who reported longer hospital and ICU stays for patients with UEIs due to the higher frequency of severe chest and abdominal injuries. Therefore, severe head, chest, and abdominal injuries seem to be a likely reason for the increased mortality rate in patients with UEIs in our study.

### Limitations

This study has several limitations due to its retrospective design, which may lead to missing information and bias, and its single-center focus, which may affect the generalizability of the findings. Also, it lacks objective measurements for functional outcomes, quality of life, and patient follow-ups. Since the study exclusively involves hospitalized patients, there is a possibility of overlooking UEF cases that were treated and discharged from the emergency department, potentially resulting in an underrepresentation of the epidemiological data. The terms UEIs and UEFs should not be used mutually/ interchangeably as each may carry different implications and ranges of damage. Therefore, we opted to use UEFs rather than UEIs. More detailed information regarding the location and potential risk factors of upper extremity injuries is needed, which limits the examination of various confounding factors in this analysis. Nevertheless, the large sample size and nationally representative data contribute to the study’s value as a significant source of information on the prevalence of hospitalized UEIs in our region. The results may exhibit gender bias, as females were presented in less than ten percent of the cases; however, this accurately reflects the pattern of hospitalized trauma patients in Qatar [14, 26].

In conclusion, most patients with upper extremity fractures were young males, primarily injured in motor vehicle crashes. The shoulder girdle, particularly clavicle and scapula/glenoid fractures, emerged as common injury sites. The study also highlighted the potential risk of pedestrian injuries, as reflected in higher injury severity, concomitant injuries, and higher mortality rates. Surgical interventions were often required for specific fractures, emphasizing the complexity of treatment decisions based on the type of injury. Future studies are needed to optimize preventive measures by incorporating insights from this study on specific injury mechanisms and patterns of upper extremity injuries.

## Data Availability

All data were presented in the manuscript and tables.

## References

[1] Wenzinger E, Rivera-Barrios A, Gonzalez G, Herrera F (2019) Trends in upper extremity injuries presenting to US emergency departments. Hand (NY) 14(3):408–412. 10.1177/1558944717735943 10.1177/1558944717735943 PMC6535948 29121783

[2] Arhami Dolatabadi A, Ebrahimzadeh N, Amini A, Shojaee M, Amiri M (2016) Epidemiology of upper extremity trauma in patients visiting the emergency department. Iran J Emerg Med 3(1):39–34. 10.22037/ijem.v3i1.11090

[3] Starnoni M, Benanti E, Acciaro AL, De Santis G (2021) Upper limb traumatic injuries: a concise overview of reconstructive options. Ann Med Surg (Lond) 27(66):102418. 10.1016/j.amsu.2021.102418 10.1016/j.amsu.2021.102418 PMC8188247 34141410

[4] Banerjee M, Bouillon B, Shafizadeh S, Paffrath T, Lefering R, Wafaisade A, German Trauma Registry Group (2013) Epidemiology of extremity injuries in multiple trauma patients. Injury 44(8):1015–1021. 10.1016/j.injury.2012.12.007. 10.1016/j.injury.2012.12.007 23287554

[5] Sharrock M (2021) The mangled extremity: assessment, decision-making, and outcomes. Acta Orthop Belg 87(4):755–760. 10.52628/87.4.22 35172444 10.52628/87.4.22

[6] Hohenberger GM, Cambiaso-Daniel J, Schwarz AM, Boukovalas S, Seibert FJ, Konstantiniuk P, Cohnert T (2020) Traumatic upper extremity injuries: analysis of correlation of mangled extremity severity score and disabilities of the arm, shoulder and hand score. Ulus Travma Acil Cerrahi Derg 26(1):95–102. English. 10.14744/tjtes.2019.44939. 10.14744/tjtes.2019.44939 31942737

[7] Joshi V, Harding GE, Bottoni DA, Lovell MB, Forbes TL (2007) Determination of functional outcome following upper extremity arterial trauma. Vasc Endovascular Surg 41(2):111–114. 10.1177/1538574406291338 10.1177/1538574406291338 17463199

[8] Allemann F, Heining S, Zelle B, Probst C, Pape HC (2019) Risk factors for complications and adverse outcomes in polytrauma patients with associated upper extremity injuries. Patient Saf Surg 4(13):7. 10.1186/s13037-019-0187-3 10.1186/s13037-019-0187-3 PMC6360674 30740144

[9] Ameri M, Aghakhani K, Ameri E, Mehrpisheh S, Memarian A (2017) Epidemiology of the upper extremity trauma in a traumatic center in Iran. Global J Health Sci 9:97–105. 10.5539/gjhs.v9n4p97 10.5539/gjhs.v9n4p97

[10] Karl JW, Olson PR, Rosenwasser MP (2015) The epidemiology of upper extremity fractures in the United States, 2009. J Orthop Trauma 29(8):e242–e244. 10.1097/BOT.0000000000000312 25714441 10.1097/BOT.0000000000000312

[11] Giustini M, de Leo A, Leti Acciaro A, Pajardi G, Mamo C, Voller F, Fadda F, Fondi G, Pitidis A (2015) Incidence estimates of hand and upper extremity injuries in Italy. Ann Ist Super Sanita 51(4):305–312. 10.4415/ANN_15_04_10 26783217 10.4415/ANN_15_04_10

[12] Polinder S, Iordens GI, Panneman MJ et al (2013) Trends in incidence and costs of injuries to the shoulder, arm, and wrist in The Netherlands between 1986 and 2008. BMC Public Health 13:531. 10.1186/1471-2458-13-531 23724850 10.1186/1471-2458-13-531 PMC3750605

[13] Hoveidaei AH, Nakhostin-Ansari A, Namdari S, Hosseini-Asl SH, Khonji MS, Selk-Ghaffari M, Pouramini A, LaPorte DM (2023) Increasing burden of upper-extremity fractures in the Middle East and North Africa (MENA): a 30-year analysis of the epidemiology and causes of injuries. J Bone Joint Surg Am. 10.2106/JBJS.23.00262 38000016 10.2106/JBJS.23.00262

[14] El-Menyar A, Mekkodathil A, Asim M, Consunji R, Strandvik G, Peralta R, Rizoli S, Abdelrahman H, Mollazehi M, Parchani A et al (2020) Maturation process and international accreditation of trauma system in a rapidly developing country. PLoS ONE 15:e0243658 33301481 10.1371/journal.pone.0243658 PMC7728290

[15] Al-Thani H, El-Menyar A, Khan NA, Consunji R, Mendez G, Abulkhair TS, Mollazehi M, Peralta R, Abdelrahman H, Chughtai T, Rizoli S (2023) Trauma quality improvement program: a retrospective analysis from a middle eastern national trauma center. Healthcare (Basel) 11(21):2865. 10.3390/healthcare11212865 37958008 10.3390/healthcare11212865 PMC10649144

[16] Singer AJ, Thode HC Jr, Hollander JE (2006) National trends in ED lacerations between 1992 and 2002. Am J Emerg Med 24(2):183–188. 10.1016/j.ajem.2005.08.021 10.1016/j.ajem.2005.08.021 16490648

[17] Briese T, Theisen C, Schliemann B, Raschke MJ, Lefering R, Weimann A (2021) Shoulder injuries in polytraumatized patients: an analysis of the TraumaRegister DGU®. Eur J Trauma Emerg Surg 47(6):1921–1930. 10.1007/s00068-020-01340-1 32221637 10.1007/s00068-020-01340-1 PMC8629800

[18] Ribak S, de Oliveira EJN, Rosolino GP, Orru P, Tietzmann A (2018) Epidemiology of traumatic injuries of the upper limbs in a university hospital. Acta Ortop Bras 26(6):370–373. 10.1590/1413-785220182606180607 30774508 10.1590/1413-785220182606180607 PMC6362678

[19] Arroyo-Berezowsky C, Quinzaños-Fresnedo J (2021) Epidemiology of hand and wrist injuries treated in a reference specialty center over a year. Acta Ortop Mex 35(5): 429–435. 10.35366/104570 35451252

[20] Ootes D, Lambers KT, Ring DC (2012) The epidemiology of upper extremity injuries presenting to the emergency department in the United States. Hand (N Y) 7(1):18–22. 10.1007/s11552-011-9383-z 23449400 10.1007/s11552-011-9383-z PMC3280373

[21] van Onselen EB, Karim RB, Hage JJ, Ritt MJ (2003) Prevalence and distribution of hand fractures. J Hand Surg Br 28(5):491–495. 10.1016/s0266-7681(03)00103-7 12954264 10.1016/s0266-7681(03)00103-7

[22] Rubin G, Peleg K, Givon A, Israel Trauma Group, Rozen N (2015) Upper extremity fractures among hospitalized road traffic accident adults. Am J Emerg Med 33(2):250–253. 10.1016/j.ajem.2014.11.048 10.1016/j.ajem.2014.11.048 25534120

[23] Siram SM, Sonaike V, Bolorunduro OB, Greene WR, Gerald SZ, Chang DC, Cornwell EE 3rd, Oyetunji TA (2011) Does the pattern of injury in elderly pedestrian trauma mirror that of the younger pedestrian? J Surg Res 167(1):14–18. 10.1016/j.jss.2010.10.007 21109262 10.1016/j.jss.2010.10.007

[24] Adili A, Bhandari M, Sprague S, Dunlop RB, Schemitsch EH (2002) Humeral shaft fractures as predictors of intra-abdominal injury in motor vehicle collision victims. Arch Orthop Trauma Surg 122(1):5–9. 10.1007/s004020100315 11995882 10.1007/s004020100315

[25] Zeelenberg ML, Den Hartog D, Halvachizadeh S, Pape HC, Verhofstad MHJ, Van Lieshout EMM (2022) The impact of upper-extremity injuries on polytrauma patients at a level 1 trauma center. J Shoulder Elbow Surg 31(5):914–922. 10.1016/j.jse.2021.10.005 34687916 10.1016/j.jse.2021.10.005

[26] El-Menyar A, El-Hennawy H, Al-Thani H, Asim M, Abdelrahman H, Zarour A, Parchani A, Peralta R, Latifi R (2014) Traumatic injury among females: Does gender matter? J Trauma Manag Outcomes 28(8):8. 10.1186/1752-2897-8-8 10.1186/1752-2897-8-8 PMC4118222 25089153

